# LncRNA *MCM3AP-AS1* serves as a competing endogenous RNA of *miR-218* to upregulate *GLUT1* in papillary thyroid carcinoma

**DOI:** 10.20945/2359-3997000000510

**Published:** 2022-08-04

**Authors:** Rui Nian, Wanjun Li, Xiang Li, Jiayu Zhang, Weihua Li, Fanfan Pan, Jing Cheng, Xin Jin

**Affiliations:** 1 Affiliated 3201 Hospital of Xi’an Jiaotong University Department of Pathology Hanzhong City PR China Department of Pathology, Affiliated 3201 Hospital of Xi’an Jiaotong University, Hanzhong City, Shaanxi Province, PR China

**Keywords:** MCM3AP-AS1, papillary thyroid cancer (PTC), GLUT1, miR-218

## Abstract

**Objective:**

*MCM3AP-AS1* has been characterized as an oncogenic long non-coding RNA (lncRNA) in several cancers including papillary thyroid cancer (PTC), but its role in PTC has not been fully elucidated. Considering the critical role of lncRNAs in cancer biology, further functional analysis of *MCM3AP-AS1* in PTC may provide novel insights into PTC management.

**Subjects and methods:**

Paired tumor and non-tumor tissues were collected from 63 papillary thyroid carcinoma (PTC) patients. Expression levels of *MCM3AP-AS1* , *miR-218* and GLUT1 in tissue samples were analyzed by qRT-PCR. Cell transfection was performed to explore the interactions among *MCM3AP-AS1* , *miR-218* and *GLUT1* . Cell proliferation assay was performed to evaluate the effects of *MCM3AP-AS1* and *miR-218* on cell proliferation.

**Results:**

*MCM3AP-AS1* accumulated to high levels in PTC tissues and was affected by clinical stage. *MCM3AP-AS1* showed a positive correlation with GLUT1 across PTC tissues. RNA interaction prediction showed that *MCM3AP-AS1* could bind to *miR-218* , which can directly target *GLUT1* . *MCM3AP-AS1* and *miR-218* showed no regulatory role regulating the expression of each other, but overexpression of *MCM3AP-AS1* upregulated *GLUT1* and enhanced cell proliferation. In contrast, overexpression of *miR-218* downregulated *GLUT1* and attenuated cell proliferation. In addition, *miR-218* suppressed the role of *MCM3AP-AS1* in regulating the expression of *GLUT1* and cell proliferation.

**Conclusions:**

*MCM3AP-AS1* may serve as a competing endogenous RNA of *miR-218* to upregulate *GLUT1* in PTC, thereby promoting cell proliferation. The *MCM3AP-AS1/miR-218/GLUT1* pathway characterized in the present study might serve as a potential target to treat PTC.

## INTRODUCTION

Papillary thyroid carcinoma (PTC) is a type of well-differentiated thyroid carcinoma and accounts for more than 80% of all thyroid carcinoma cases ( 1 ). Due to the well-differentiated characteristic, PTC usually grows slowly in local region ( 2 ). Therefore, most PTC patients can be treated with optimized surgery in combination with levothyroxine suppression therapy and/or radioiodine treatment, and the treatment outcomes are generally satisfactory ( 3 ). However, metastasis may occur in extreme cases, and the survival of patients with metastatic PTC is still poor ( 4 ). In addition, the incidence rate of PTC has been rapidly increasing over the past several decades ( 5 , 6 ). Therefore, novel preventive and therapeutic approaches are still needed.

Accelerated glucose metabolism provides energy for the growth of tumors, and inhibition of glucose metabolism is considered as a potential therapeutic approach for cancer therapy ( 7 ). *Glucose transporter 1* ( *GLUT1* ) plays critical roles in the early steps of glucose metabolism by mediating the transportation of glucose across cell membrane ( 8 ). *GLUT1* is usually upregulated in cancers, and high expression levels of *GLUT1* predict poor survival of PTC patients ( 9 ). Certain tumor suppressive miRNAs, such as *miR-218* , can suppress cancer development by targeting *GLUT1* ( 10 ). Extensive studies have been performed to characterize the long non-coding RNAs (lncRNAs)/mRNAs/miRNAs related competing endogenous RNAs (ceRNAs) network in various carcinoma cells ( 11 ). Generally, lncRNAs can serve as the ceRNAs of mature miRNAs in cytoplasm to suppress their role in inhibiting gene expression, thereby by indirectly regulating the expression of downstream tumor suppressors and oncogenes ( 11 ). Our bioinformatics analysis showed that miR-218 could form a strong base pairing with lncRNA (>200 nt) *MCM3AP-AS1* (2539 bp, Accession: KJ903636.1). *MCM3AP-AS1* was reported to play an oncogenic role in many cancers ( 12 , 14 ). In most cases, *MCM3AP-AS1* upregulates the expression of oncogenes by serving as the ceRNA of miRNAs. In papillary thyroid cancer (PTC), *MCM3AP-AS1* was reported to promote proliferation and invasion of cancer cells through regulating the *miR-211-5p/SPARC* axis ( 15 ) . However, its role in PTC has not been fully elucidated. We speculated that *MCM3AP-AS1* may serve as the ceRNA of *miR-218* to regulate *GLUT1* , thereby participating in PTC. This study was therefore carried out to investigate the interactions among *MCM3AP-AS1* , *miR-218* and *GLUT1* in PTC. *MCM3AP-AS1* may serve as a ceRNA of *miR-218* to upregulate *GLUT1* in PTC, thereby promoting cell proliferation.

## SUBJECTS AND METHODS

### PTC patients and specimen collection

This study enrolled a total of 63 PTC patients (21 males and 44 females, age range from 21 to 55 years old, mean age 35.5 ± 6.7 years old) selected from 144 PTC patients who were admitted at the 3201 Medical College Affiliated Hospital between April 2016 and May 2019. Inclusion criteria: 1) newly diagnosed cases of PTC; 2) complete medical record of patients; 3) therapies were not initiated. Exclusion criteria: 1) recurrent PTC; 2) patients with other clinical disorders; 3) initiated therapies. This study was approved by the Ethics Committee of aforementioned hospital (Ethics approval no. IUR-TYC2432). All patients were informed of the details of the experimental design and signed the written informed consent. All patients were subjected to biopsy that was performed under the guidance of ultrasound using a fine needle. PTC tumor tissues and non-tumor tissues were collected from each patient by dissecting biopsy. All tissue specimens were confirmed by histopathological exams.

### Vectors and miRNA mimic

PcDNA3.1 vector (GenePharma, Shanghai, China) was used to construct vectors expressing *MCM3AP-AS1* and *GLUT1* by inserting the full-length cDNA or *MCM3AP-AS1* (2539 bp), and *GLUT1* (1479 bp) into vector. Negative control (NC) miRNA and *miR-218* mimic were also purchased from GenePharma.

### Cell line and transient cell transfection

PTC cell lines IHH-4 (Stage IV) and MDA-T120 (stage IV) were purchased from ATCC (Manassas, VA, USA) and used as the cell model of PTC. Cells were cultivated in a mixture containing 10% FBS and 90% DMEM at 37 °C with 5% CO_2_ and 95% humidity.

To perform transfection, cells were harvested at 80% confluence. Cells were counted and 40 nM miRNAs (NC miRNA was used as NC group) or 10 nM vectors (empty vector was used as NC group) were transfected into 3 × 10^6^ IHH-4 and MDA-T120 cells through transient transfections using Lipofectamine 2000 (GenePharma). Cells were incubated with the transfection mixture for 6 h. After that, cells were washed with fresh cell culture medium. Cells were harvested at 24 h post-transfection to perform the subsequent experiments. Untransfected cells were used as the Control (C) cells.

### Dual-luciferase reporter assay

Dual-luciferase reporter assay was performed to investigate the interaction between *MCM3AP-AS1* and *miR-218* in IHH-4 cells using the Promega Dual-Luciferase™ Reporter (DLR™) Assay System (Promega) following the manufacturer’s instructions. The binding site of *miR-218* on *MCM3AP-AS1* was cloned into pGL3-Promoter Vector at 5’ upstream of the luciferase gene. Two different combinations of transfection were performed: 1) *MCM3AP-AS1* + *miR-218* -WT; 2) *MCM3AP-AS1* + *miR-218-MUT* (mutant site was marked in Figure 2D ). Firefly luminescence was used to normalize the renilla luminescence.

### qRT-PCR

Ribozol (Sigma-Aldrich) was used to extract total RNAs from ground tissue samples (0.03 g) and IHH-4 cells (3 × 10^5^). RNA samples were precipitated and washed using 85% ethanol to harvest miRNAs. RNA samples were digested with DNase I at 37 °C for 60 min to remove genomic DNA. Prior to the following applications, RNA samples were stored at -80 °C. Reverse transcriptions were performed using the TruScript Reverse Transcriptase Kit (Norgenbiotek). To measure the expression levels of *MCM3AP-AS1* and *GLUT1* , KAPA SYBR FAST qPCR Kit (Roche) was used to prepare all qPCR reaction mixtures. *GAPDH* was used as the endogenous control. B-actin was also used as an endogenous control and similar results were obtained. To measure the expression levels of miR-218, All-in-One™ miRNA qRT-PCR Detection Kit (Genecopoeia) was used to perform the addition of poly (A), miRNA reverse transcriptions and qPCR assays. In qPCR assays, U6 was used as the endogenous control. Primer amplification efficiency in all cases was between 98.8 and 99.7%. qPCR reactions were performed in 3 replicates. The relative expression levels were calculated using the 2^-ΔΔCT^ method. For the comparison of expression levels among multiple genes, the gene in the sample with the biggest ΔCT value was set to value “1”, and all other samples were normalized to this sample to calculate the relative expression levels. Primer sequences were: 5’-CTGCTAATGGCAACACTGA-3’ (forward) and 5’-AGGTGCTGTCTGGTGGAGA-3’ (reverse) for MCM3AP-AS1; 5’-CAGGAGGCATTGCTGATGAT-3’ (forward) and 5’-GAAGGCTGGGGCTCATTT-3’ (reverse) for GAPDH; 5’-CACCATTGGCAATGAGCGGTTC-3’ (forward) and 5’-AGGTCTTTGCGGATGTCCACGT-3’ (reverse) for β-actin; 5’-GAGCCTGAGCGGGAGAGC-3’ (forward) and 5’-GACCCGTCAGCTTCTTGC-3’ (reverse) for GLUT1; 5’-TTCGGCAGCACATATACTAAAAT-3’ (forward) and 5’-CGCTTCACGAATTTGCGTGTC-3’ (reverse) for U6; 5’-TTGTGCTTGATCTAACCA-3’ (forward) and reverse poly(T) for miR-218.

### Western blot analysis

The expression levels of GLUT1 protein in IHH-4 cells were measured at 24 h post-transfection. Total proteins were extracted from 3 × 10^5^ IHH-4 cells using RIPA solution (GenePharma). Protein samples were quantified using BCA assay (GenePharma). Protein samples were incubated in boiling water for 12 min for denaturation, followed by electrophoresis using 10% SDS-PAGE gel (80 min at 100 V). Proteins were then transferred to PVDF membranes (20 min at 45 V), and PBS containing 5% non-fat milk was used to block the membranes at 22 °C for 2 h. Next, rabbit anti- *GAPDH* (1:1,800, ab37168, Abcam) or anti- *GLUT1* (1:2,000, ab15309, Abcam) primary antibodies were used to incubate the membranes at 4 °C for 16 h, followed by incubation with goat HRP (IgG) secondary antibody (1:1,800; ab6721; Abcam) at 22 °C for 2 h. Pierce ECL Western Blotting Substrate (Thermo Fisher Scientific) was then used to incubate the membranes at 22 °C for 1 min. Images were taken using myECL Imager (Thermo Fisher Scientific) after exposure for 10 min. Image J v1.46 software was used to process all data.

### Cell proliferation analysis

The effects of cell transfection on the proliferation of IHH-4 and MDA-T120 cells were assessed by CCK-8 assay. Briefly, 1 ml aforementioned cell culture medium was used to re-suspend cell pellets (containing 3 × 10^4^ cells) to prepare single cell suspensions. Cells were cultivated in cell culture plate (96-well) under the aforementioned conditions. Each transfection group included 3 replicate wells. Each well was added with 10 μL CCK-8 solution (Sigma-Aldrich) at 4 h before the termination of cell culture. Following that, 10 μL DMSO was added, and OD values at 450 nm were measured.

### FISH

Fluorescence *in situ* hybridization (FISH) was performed as previously described ( 16 ). Briefly, IHH-4 cells grown on the slides were washed with PBS and then fixed in 4% paraformaldehyde. After treatment with protease reagent, the slides were incubated with prehybridization buffer at 40 °C for 4 h, and then hybridized with digoxin-labeled probe at 40 °C overnight. FISH probe sequence of *MCM3AP-AS1* was 5’ (digoxigenin)-TAATGTCTGTTATCATGGTATCTGTGGGTCAGGAATCCAGGTG-3’.

### Statistical analysis

All experiments were performed in 3 biological replicates. Mean values were calculated and used for data analyses. Differences between two types of tissue from PTC patients were compared by paired t-test. Differences among multiple patient or cell groups were compared by ANOVA (one-way) combined with Tukey test. Pearson’s correlation coefficient was used to analyze correlations. *P* < 0.05 was considered statistically significant.

## RESULTS

### *MCM3AP-AS* 1 was upregulated in PTC and affected by clinical stages

**Figure 1:**
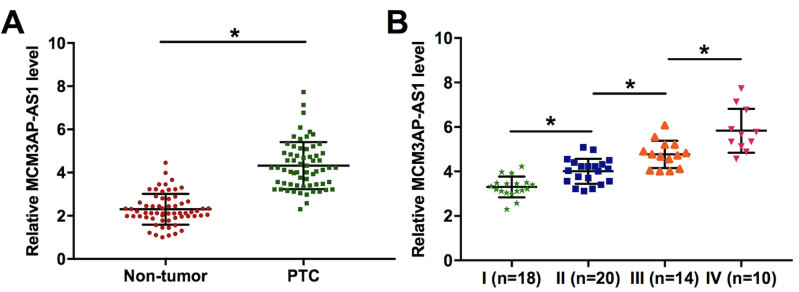
*MCM3AP-AS1* was upregulated in PTC and affected by clinical stages. The expression of *MCM3AP-AS1* in both PTC and non-tumor tissues were measured by qRT-PCR. The expression levels of *MCM3AP-AS1* were compared between two types of tissue by performing paired t-test ( **A** ). ANOVA (one-way) combined with Tukey test was used to compare the expression levels of *MCM3AP-AS1* among patients at different clinical stages ( **B** ). Mean values of 3 biological replicates were presented, *, *p* < 0.05.

The expression levels of *MCM3AP-AS1* in both PTC and non-tumor tissues were measured by qRT-PCR. The sample with the lowest expression level was set to value “1”. Other samples were normalized to this sample. Compared with non-tumor tissues, the expression levels of *MCM3AP-AS1* were significantly increased in PTC tissues ( Figure 1A , *p* < 0.05). Based on clinical findings, the 63 patients were staged according to AJCC staging system. There were 18, 20, 17 and 8 cases at clinical stage I-IV, respectively. As shown in Figure 1B , the expression levels of *MCM3AP-AS1* were significantly increased with the increasing of clinical stage ( *p* < 0.05).

### *MCM3AP-AS1* may bind to miR-218, but no interaction between *MCM3AP-AS1* and *miR-218* was observed in IHH-4 cells

**Figure 2:**
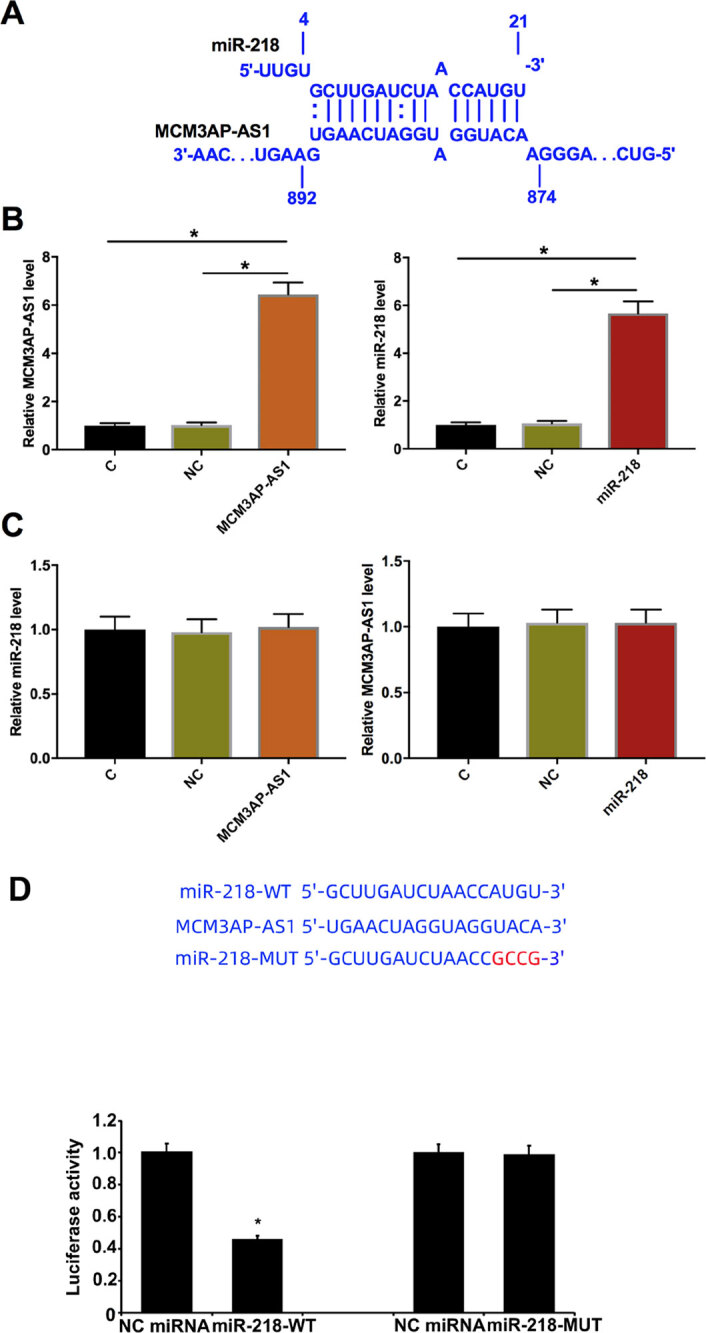
*MCM3AP-AS1* might bind to *miR-218* , but no interaction between *MCM3AP-AS1* and *miR-218* was observed in IHH-4 cells. We performed RNA interaction predictions using http://rna.informatik.uni-freiburg.de/IntaRNA/Input.jsp . *MCM3AP-AS1* and *miR-218* might form strong base pairing ( **A** ). IHH-4 cells were transfected with *MCM3AP-AS1* expression vector or *miR-218* mimic. Overexpression of *MCM3AP-AS1* and *miR-218* was confirmed by qRT-PCR at 24 h post-transfection ( **B** ). The interaction between *MCM3AP-AS1* and *miR-218* was analyzed by qRT-PCR ( **C** ). Mean values of 3 biological replicates were presented, *, p < 0.05. ( **D** ) The luciferase result between *MCM3AP-AS1* + *miR-218* -MUT or *MCM3AP-AS1* + *miR-218* -WT. Mean values of 3 biological replicates were presented, *, p < 0.05.

FISH was performed to analyze the subcellular localization of *MCM3AP-AS1* , and the results showed that *MCM3AP-AS1* was localized in cytoplasm (supplemental data-1). RNA interaction was predicted using the online tool http://rna.informatik.uni-freiburg.de/IntaRNA/Input.jsp . It showed that *MCM3AP-AS1* and *miR-218* may form multiple base pairs ( Figure 2A ). Although *miR-218* may bind to multiple sites of *MCM3AP-AS1* , only the one with the highest affinity was present. The binding energy was -13.26070 kcal/mol, indicating a strong base pairing between them. To further evaluate the interaction between *MCM3AP-AS1* and *miR-218* , IHH-4 cells were transfected with *MCM3AP-AS1* expression vector or *miR-218* mimic. Compared with NC (NC miRNA or empty pcDNA3.1 vector), the expression levels of *MCM3AP-AS1* and *miR-218* were significantly increased ( Figure 2B , *p* < 0.05). However, overexpression of *MCM3AP-AS1* and *miR-218* did not affect the expression of each other ( Figure 2C , *p* < 0.05). On the other hand, luciferase assay results indicated the sponge relationship between *MCM3AP-AS1* and *miR-218* . To perform dual luciferase activity assay, the binding site of miR-218 on *MCM3AP-AS1* was cloned into pGL3-Promoter Vector at 5’ upstream of the luciferase gene. Overexpression of *miR-218* -WT could attenuate the luciferase activity of *MCM3AP-AS1* , but *miR-218* -MUT could not ( Figure 2D , *p* < 0.05). The principle is that the binding of *miR-218* -WT could downregulate the expression of downstream luciferase gene, leading to reduced luciferase activity.

### The expression of *MCM3AP-AS1* was correlated with the expression of *GLUT1* in PTC tissues

**Figure 3:**
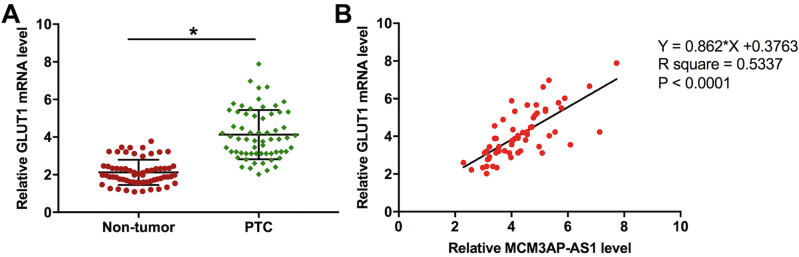
The expression of *MCM3AP-AS1* was correlated with the expression of GLUT1 in PTC tissues. The expression of GLUT1 (a downstream target of miR-218) in both PTC and non-tumor tissues were measured by qRT-PCR. The expression levels of *GLUT1* were compared between two types of tissue by performing paired t-test ( **A** ). The correlation between *GLUT1* mRNA and *MCM3AP-AS1* was analyzed by performing Pearson’s Correlation Coefficient ( **B** ). Mean values of 3 biological replicates were presented, *, *p* < 0.05.

The expression levels of *GLUT1* (a downstream target of miR-218) in both PTC and non-tumor tissues were measured by qRT-PCR. Compared with non-tumor tissues, the expression levels of *GLUT1* were significantly higher in PTC tissues ( Figure 3A , *p* < 0.05). The correlation between the expression of *GLUT1* and *MCM3AP-AS1* was analyzed by performing Pearson’s Correlation Coefficient. It was observed that the expression of *GLUT1* was significantly and positively correlated with the expression of *MCM3AP-AS1* ( Figure 3B ).

### *MCM3AP-AS1* regulated the expression of *GLUT1* in IHH-4 cells through miR-218

**Figure 4:**
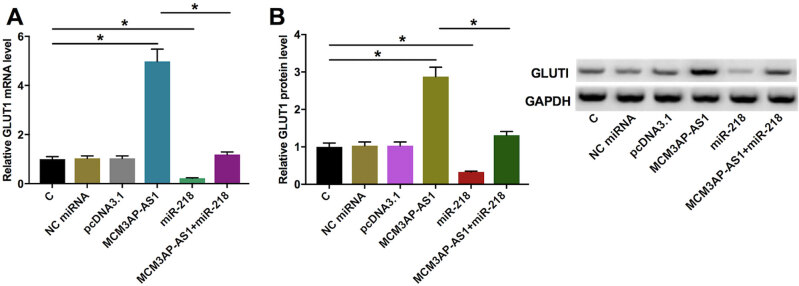
*MCM3AP-AS1* regulated the expression of *GLUT1* in IHH-4 cells through *miR-218* . qRT-PCR and Western blot were performed to analyze the effects of overexpression of *MCM3AP-AS1* on GLUT1 at both mRNA ( **A** ) and protein ( **B** ) levels. Mean values of 3 biological replicates were presented, *, *p* < 0.05. Check the additional file for the original, uncropped gels or blots. The original blots were presented in Figure 1 .

Western blot analysis showed that overexpression of *MCM3AP-AS1* significantly upregulated *GLUT1* at both mRNA ( Figure 4A ) and protein ( Figure 4B ) levels compared with NC (NC miRNA or empty pcDNA3.1 vector) ( *p* < 0.05) (original blots imagines were shown in supplementary Figure 1 ). However, overexpression of *miR-218* significantly downregulated *GLUT1* at both mRNA and protein levels. In addition, co-transfection experiment showed that overexpression of *miR-218* attenuated the effects of overexpression of *MCM3AP-AS1* on the expression of *GLUT1* .

### *MCM3AP-AS1* promoted the proliferation of IHH-4 and *MDA-T120* cells through *miR-218* and GLUT1

**Figure 5:**
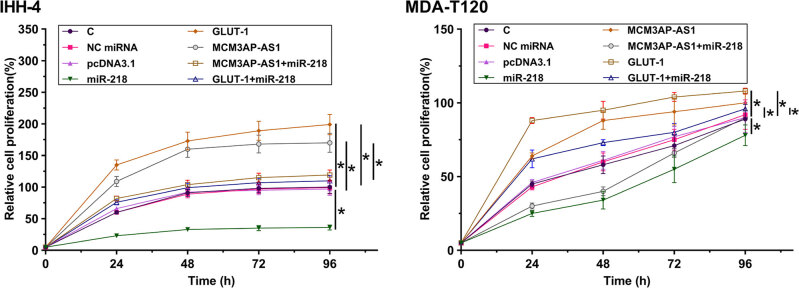
*MCM3AP-AS1* promoted the proliferation of IHH-4 and MDA-T120 cells through *miR-218* and GLUT1. CCK-8 assay was performed to analyze the effects of *MCM3AP-AS1, miR-218, GLUT1, MCM3AP-AS1* + *miR-218* and *GLUT1* + *miR-218* overexpression on the proliferation of IHH-4 and MDA-T120 cells at 24 h post-transfection. Mean values of 3 biological replicates were presented, *, *p* < 0.05.

CCK-8 assay was performed to evaluate the effects of overexpression of *MCM3AP-AS1* , *miR-218* and *GLUT1* on the proliferation of IHH-4 and MDA-T120 cells. Compared with NC (NC miRNA or empty pcDNA3.1 vector), overexpression of *MCM3AP-AS1* and *GLUT1* increased the proliferation rate of IHH-4 and MDA-T120 cells ( Figure 5 , *p* < 0.05). However, overexpression of *miR-218* attenuated the proliferation of IHH-4 and MDA-T120 cells ( Figure 5 , *p* < 0.05). In addition, co-transfection experiment showed that overexpression of miR-218 attenuated the effects of overexpression of *MCM3AP-AS1* and *GLUT1* on the proliferation of IHH-4 and MDA-T120 cells ( Figure 5 , *p* < 0.05).

## DISCUSSION

In the present study, we found that *MCM3AP-AS1* was upregulated in PTC and might upregulate *GLUT1* by sponging *miR-218* to promote the proliferation of PTC cells.

The functions of *MCM3AP-AS1* have only been investigated in a few cancers (12,13,15). In liver cancer, *MCM3AP-AS1* is upregulated and can regulate the *miR-194-5p/FOXA1* axis to promote tumor growth ( 12 ). In glioblastoma, *MCM3AP-AS1* is also upregulated and negatively regulates the expression of *miR-211* , which in turn activates the PI3K/AKT and ERK1/2 signaling pathways through KLF5 ( 13 ). These studies revealed the oncogenic function of *MCM3AP-AS1* in cancer biology. Besides, *MCM3AP-AS1* was reported to be upregulated in PTC and it promotes proliferation and invasion of cancer cells through regulating the *miR-211-5p/SPARC* axis in PTC ( 15 ). In this study, we confirmed that *MCM3AP-AS1* was upregulated in PTC, and overexpression of *MCM3AP-AS1* resulted in the increased proliferation of PTC cells. Therefore, our data suggest that *MCM3AP-AS1* plays an oncogenic role in PTC by promoting cancer cell proliferation.

*MiR-218* is a well-characterized tumor suppressive miRNA that suppresses cancer development and progression by affecting cancer cell behaviors, such as proliferation ( 17 , 18 ). Downregulation of *miR-218* is closely correlated with the progression of PTC ( 19 ). Consistently, our study also observed the inhibitory effects of *miR-218* on PTC cell proliferation. We found that *miR-218* could form strong base paring with *MCM3AP-AS1* . However, overexpression of *miR-218* in PTC cells did not affect the expression of *MCM3AP-AS1* . It is known that lncRNAs may sponge miRNAs to reduce their effects on downstream targets ( 20 ). It has been reported that *miR-218* can target *GLUT1* to inhibit bladder cancer ( 10 ). In PTC, increased expression levels of *GLUT1* could promote tumor growth, leading to poor prognosis ( 9 ). This study also reported the downregulation of *GLUT1* in PTC cells after overexpression of *miR-218* . Therefore, *miR-218* may also target *GLUT1* in PTC. In addition, overexpression of *MCM3AP-AS1* resulted in upregulation of *GLUT1* . Our data supported the speculation that *MCM3AP-AS1* might sponge *miR-218* to upregulate *GLUT1* .

It is worth noting that, based on the predicted secondary structure of *MCM3AP-AS1* by RNA fold ( http://rna.tbi.univie.ac.at/cgi-bin/RNAWebSuite/RNAfold.cgi ), the binding site of *miR-218* on *MCM3AP-AS1* was mapped to a loop. However, it is known that bases in loop regions may also form noncanonical base pairs with biological functions ( 21 ). Future studies are still needed to further elucidate the interaction between *miR-218* and *MCM3AP-AS1* .

In conclusion, *MCM3AP-AS1* plays an oncogenic role in PTC possibly by sponging *miR-218* to upregulate *GLUT1* and promote cancer cell proliferation. The *MCM3AP-AS1/miR-218/GLUT1* axis may serve a potential target to treat PTC.

## Figures and Tables

**Figure:**
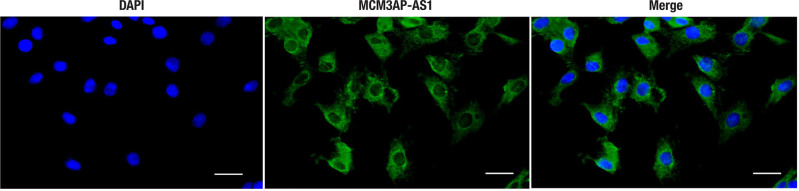
Supplement data 1. Subcellular location of MCM3AP-AS1 in IHH-4 cells. RNA FISH was employed to detect the MCM3AP-AS1 expression in the IHH-4 cells. DAPI fluorescence indicate the nuclei(left). MCM3AP-AS1 was detected in cytoplasm (middle). The merged showed in left. scale bar = 100 μm.

## References

[1] Teng L, Deng W, Lu J, Zhang J, Ren X, Duan H (2017). Hobnail variant of papillary thyroid carcinoma: molecular profiling and comparison to classical papillary thyroid carcinoma, poorly differentiated thyroid carcinoma and anaplastic thyroid carcinoma. Oncotarget.

[2] Zhang H, Teng X, Liu Z, Zhang L, Liu Z (2015). Gene expression profile analyze the molecular mechanism of CXCR7 regulating papillary thyroid carcinoma growth and metastasis. J Exp Clin Cancer Res.

[3] Grant CS (2015). Recurrence of papillary thyroid cancer after optimized surgery. Gland Surg.

[4] Mauri G, Cova L, Ierace T, Baroli A, Di Mauro E, Pacella CM (2016). Treatment of Metastatic Lymph Nodes in the Neck from Papillary Thyroid Carcinoma with Percutaneous Laser Ablation. Cardiovasc Intervent Radiol.

[5] Pellegriti G, Frasca F, Regalbuto C, Squatrito S, Vigneri R (2013). Worldwide increasing incidence of thyroid cancer: update on epidemiology and risk factors. J Cancer Epidemiol.

[6] Gimm O, Castellone MD, Hoang-Vu C, Kebebew E (2011). Biomarkers in thyroid tumor research: new diagnostic tools and potential targets of molecular-based therapy. J Thyroid Res.

[7] Shen CT, Wei WJ, Qiu ZL, Song HJ, Luo QY (2016). Afamin promotes glucose metabolism in papillary thyroid carcinoma. Mol Cell Endocrinol.

[8] Freemerman AJ, Johnson AR, Sacks GN, Milner JJ, Kirk EL, Troester MA (2014). Metabolic reprogramming of macrophages: glucose transporter 1 (GLUT1)-mediated glucose metabolism drives a proinflammatory phenotype. J Biol Chem.

[9] Chai YJ, Yi JW, Oh SW, Kim YA, Yi KH, Kim JH (2017). Upregulation of SLC2 (GLUT) family genes is related to poor survival outcomes in papillary thyroid carcinoma: Analysis of data from The Cancer Genome Atlas. Surgery.

[10] Li P, Yang X, Cheng Y, Zhang X, Yang C, Deng X (2017). MicroRNA-218 Increases the Sensitivity of Bladder Cancer to Cisplatin by Targeting Glut1. Cell Physiol Biochem.

[11] Zhang Y, Xu Y, Feng L, Li F, Sun Z, Wu T (2016). Comprehensive characterization of lncRNA-mRNA related ceRNA network across 12 major cancers. Oncotarget.

[12] Wang Y, Yang L, Chen T, Liu X, Guo Y, Zhu Q (2019). A novel lncRNA MCM3AP-AS1 promotes the growth of hepatocellular carcinoma by targeting miR-194-5p/FOXA1 axis. Mol Cancer.

[13] Yang C, Zheng J, Xue Y, Yu H, Liu X, Ma J (2018). The Effect of MCM3AP-AS1/miR-211/KLF5/AGGF1 Axis Regulating Glioblastoma Angiogenesis. Front Mol Neurosci.

[14] Wu J, Lv Y, Li Y, Jiang Y, Wang L, Zhang X (2020). MCM3AP-AS1/miR-876-5p/WNT5A axis regulates the proliferation of prostate cancer cells. Cancer Cell Int.

[15] Liang M, Jia J, Chen L, Wei B, Guan Q, Ding Z (2019). LncRNA MCM3AP-AS1 promotes proliferation and invasion through regulating miR-211-5p/SPARC axis in papillary thyroid cancer. Endocrine.

[16] Hu YP, Jin YP, Wu XS, Yang Y, Li YS, Li HF (2019). LncRNA-HGBC stabilized by HuR promotes gallbladder cancer progression by regulating miR-502-3p/SET/AKT axis. Mol Cancer.

[17] Tie J, Pan Y, Zhao L, Wu K, Liu J, Sun S (2010). MiR-218 inhibits invasion and metastasis of gastric cancer by targeting the Robo1 receptor. PLoS Genet.

[18] Alajez NM, Lenarduzzi M, Ito E, Hui AB, Shi W, Bruce J (2011). MiR-218 suppresses nasopharyngeal cancer progression through downregulation of survivin and the SLIT2-ROBO1 pathway. Cancer Res.

[19] Guan H, Wei G, Wu J, Fang D, Liao Z, Xiao H (2013). Down-regulation of miR-218-2 and its host gene SLIT3 cooperate to promote invasion and progression of thyroid cancer. J Clin Endocrinol Metab.

[20] Chen L, Hu N, Wang C, Zhao H, Gu Y (2018). Long non-coding RNA CCAT1 promotes multiple myeloma progression by acting as a molecular sponge of miR-181a-5p to modulate HOXA1 expression. Cell Cycle.

[21] Wu J, Leontis NB, Zirbel CL, Bisaro DM, Ding B (2019). A three-dimensional RNA motif mediates directional trafficking of Potato spindle tuber viroid from epidermal to palisade mesophyll cells in Nicotiana benthamiana. PLoS Pathog.

